# Exploring the dominant features and data-driven detection of polycystic ovary syndrome through modified stacking ensemble machine learning technique

**DOI:** 10.1016/j.heliyon.2023.e14518

**Published:** 2023-03-16

**Authors:** Sayma Alam Suha, Muhammad Nazrul Islam

**Affiliations:** Department of Computer Science and Engineering, Military Institute of Science and Technology, Dhaka, Bangladesh

**Keywords:** Polycystic ovary syndrome (PCOS), Dominant features, Machine learning classification, Stacking ensemble technique

## Abstract

Polycystic ovary syndrome (PCOS) is the most frequent endocrinological anomaly in reproductive women that causes persistent hormonal secretion disruption, leading to the formation of numerous cysts within the ovaries and serious health complications. But the real-world clinical detection technique for PCOS is very critical since the accuracy of interpretations being substantially dependent on the physician's expertise. Thus, an artificially intelligent PCOS prediction model might be a feasible additional technique to the error prone and time-consuming diagnostic technique. In this study, a modified ensemble machine learning (ML) classification approach is proposed utilizing state-of-the-art stacking technique for PCOS identification with patients' symptom data; employing five traditional ML models as base learners and then one bagging or boosting ensemble ML model as the meta-learner of the stacked model. Furthermore, three distinct types of feature selection strategies are applied to pick different sets of features with varied numbers and combinations of attributes. To evaluate and explore the dominant features necessary for predicting PCOS, the proposed technique with five variety of models and other ten types of classifiers is trained, tested and assessed utilizing different feature sets. As outcomes, the proposed stacking ensemble technique significantly enhances the accuracy in comparison to the other existing ML based techniques in case of all varieties of feature sets. However, among various models investigated to categorize PCOS and non-PCOS patients, the stacking ensemble model with ‘Gradient Boosting’ classifier as meta learner outperforms others with 95.7% accuracy while utilizing the top 25 features selected using Principal Component Analysis (PCA) feature selection technique.

## Highlights

• Proposed a modified stacking ensemble ML classifier to detect PCOS from patient symptoms.
• Applied 3 feature selection methods to extract sets with varying numbers & mixtures of attributes.
• Proposed methodology & existing ML approaches are trained & tested with different feature sets.
• Examined the essential attributes and efficacy of the suggested ensemble methodology.
• The proposed classifier outperforms with top 25 dominant features picked as per PCA technique.

## 1 Introduction

Polycystic ovary syndrome (PCOS) is amongst the most prevalent endocrinological disorders [1], [2] which is typically caused by an abnormal increase of male hormone known as androgen hormone in female body, producing a long-term disturbance in hormonal levels and, as a result, impacting negatively in normal ovarian processes, leading to formation of many cysts inside the ovary [3]. It is a diverse and heterogeneous condition which can be predicted through observing various signs in female body such as hyperandrogenism with acne, hirsutism, and alopecia; anovulation with menstrual irregularities, oligomenorrhea, amenorrhea; polycystic ovarian morphology, among many others [4], [5]. According to epidemiological research [6], PCOS is found to yield a number of detrimental life-threatning impacts that are prevalent in PCOS patients, with 44–70% women suffering from various critical side effects as well as affecting one in every ten premenopausal reproductive female throughout the world. This condition has been linked to a variety of metabolic and psychological illnesses that reduces the quality of a healthier lifestyle; including the principal cause of anovulation and irregular menstrual cycles, hormonal imbalance, type 2 diabetes, hyperandrogenism (excessive presence of the male sex hormones), insulin resistance, sudden obesity, thyroid irregularities, increased mental breakdown, sexual frustration, and so on [7], [8], [9]. Women with PCOS are more likely to develop endometrium and ovarian cancer, both of which can be fatal if not diagnosed in time [10], [11]. Thus, the most widespread endocrinological disorder PCOS is associated with a wide spectrum of symptoms and comorbidities. Recent studies indicate that if a well-standardized diagnosis technique can be utilized to detect PCOS early on, the disease can be treated with a healthy diet as well as suitable, symptom-oriented, long-term, and dynamic therapies [12].

However, due to the wide range of symptoms associated with PCOS and the existence of a variety of concomitant gynecological problems, PCOS becomes extremely difficult for physicians to accurately identify at an early phase [13]. Also, the effective identification of PCOS necessitates a lot of clinical test evaluations by qualified healthcare providers, which is sometimes unattainable in areas where expert physicians and resources are scarce. As a result, numerous young women go unnoticed and untreated as they do not have any easier way to detect their condition other than visiting expert clinicians; subsequently experience the adverse consequences of this devastating ailment, specially in the rural areas of developing and least developed countries. Thus, to address this challenge, a variety of computational algorithms have been suggested to forecast PCOS in patients intelligently based on their symptoms and test result. But, the conventional machine learning algorithms which despite their major triumphs, may fail to generate satisfactory results when working with too many attributes and underlying mechanism of complicated data, such as unbalanced, high-dimensional, noisy data, and so on [14]. In such cases, the ensemble machine learning method can be a promising state-of-the-art solution that combines multiple typical machine learning techniques to generate weak predictive results based on attributes retrieved through a variety of data projections from the dataset, and then integrates those results with diverse mechanisms to achieve better forecasting results than any individual algorithm [15]. A method of ensemble learning known as stacking ensemble takes into account diverse weak learners, trains them concurrently, and then combines them by training a meta-model to produce a forecast based on the results of a variety of weaker models [16]. However, it has been observed that using stacking ensemble methodologies to anticipate various disease outbreaks or predictions is superior to using traditional techniques, but rarely this technique has been explored to predict PCOS. Moreover, few studies have focus on exploring the minimal yet optimal features to predict PCOS effectively using various feature engineering techniques.

Therefore, the purpose of this research is to explore several traditional as well as ensemble types of machine learning classifiers to predict PCOS and also to propose an ensemble machine learning classifier based on stacking approach that employs the minimal and optimal amount of prioritized features for more efficiently detecting PCOS through patients' symptoms and test result dataset. The key contributions which has been done to acquire the objective of this research work are listed hereafter.

• An ensemble machine learning classifier based on the stacking state-of-the-art technique has been proposed, trained and tested where five types of traditional machine learning classifiers (Logistic Regression, Support Vector Machine, Decision Tree, K-Nearest Neighbour and Naive Bayes) have been used as the weak learners with one strong meta learner to classify the dataset between PCOS and non-PCOS criteria. One from five different kinds of boosting or bagging ensemble classifier (Adaptive Boosting, Categorical Boosting, eXtreme Gradient Boosting, Gradient Boosting, Random Forest Classifier) have been employed and evaluated as the meta learner of the model with an aim to explore the best performing stacked ensemble model in this scenario.
• For exploring the dominant features required for predicting PCOS, three different types of feature selection techniques (Chi-Square, Principal Component Analysis, Recursive Feature Elimination) have been employed here. Each feature selection techniques select the different sets of features with different numbers and combinations of attributes from the dataset employing their own feature prioritization methods which are then applied to the machine learning classifiers to detect PCOS.
• To validate the efficacy and potency of the proposed technique, other ten types of classifiers are also employed to attain the same objective which include five types of conventional classifiers (Support Vector Machine, Logistic Regression, Decision Tree, K-Nearest Neighbour, Naive Bayes), one bagging ensemble classifier (Random Forest) and four types boosting ensemble classifiers (Gradient Boosting, eXtreme Gradient Boosting, Adaptive Boosting, Categorical Boosting). Then a meticulous comparative performance analysis has been conducted between the traditional classifiers, bagging and boosting ensemble classifiers and the proposed stacking ensemble models through different performance parameters utilizing different sets of features obtained from feature selection techniques.

The remaining sections of the article are structured as follows: Section 2 presents the background study; the materials and methodology that have been employed in this study are demonstrated in Section 3; the result analysis with comparative findings is discussed in Section 4; and lastly, Section 5 and Section 6 contain discussion and conclusion that highlights the study's key findings along with its comparison with previous works, benefits, limitations, and future goals.

## 2 Background study

PCOS has been linked to a number of disorders resulting in diverse symptoms in patients' bodies compared to normal ovulatory women, including type-2 diabetes, cardiovascular anomalies, hypertension, dyslipidemia, insulin resistance, increased Endometrium thickness and so on [17], [18], [19]. Furthermore, PCOS also causes a variation in the range of hormonal secretion such as luteinizing hormone (LH), Follicle-stimulating hormone (FSH), Anti-Müllerian Hormone (AMH) etc. [20], [21]. Additionally, some more indicators are identified to be strongly associated with PCOS including undesirable facial/body hair, accelerated hair loss, dark spots on the skin, higher BMI, obesity and abdominal obesity with increased hip ratio, dietary habits with excessive fast food intake etc. [22], [23], [24]. As a result, the standard clinical detection approach for PCOS is very critical, and also the accuracy as well as reliability of this anomaly identification and interpretations is heavily reliant on the physician's competence in this context [25].

Thus, number of studies have been conducted to investigate computer-assisted PCOS detection techniques, which offer substantial advantages such as rapid identification of the condition in the shortest time frame with the least amount of diagnostic error and human effort. [26]. With the massive expansion of healthcare data and utilization of information technology, machine learning techniques are being one of the most widely used, efficient, and promising predictive strategies, which can analyze and retrieve key information from immense amounts of heterogeneous clinical data in order to detect diseases intelligently [27], [28]. Recently researchers also have applied various machine learning techniques in this context to detect PCOS condition from patient's symptom dataset.

For example, to categorize between PCOS and non-PCOS criteria, Danaei et al. [29] employed Extra Tree, Adaptive Boosting (AdaBoost), Bagging Ensemble with Random Forest and Multi-Layer Perceptron (MLP) classification models which were then evaluated through performance parameters using the reduced subgroups of features obtained by filter, embedded, and wrapper feature extraction techniques. For feature selection, Nasim et al. [30] presented an improved chi-squared (CS-PCOS) mechanism and they then conducted a performance comparison analysis of ten hyper-parametrized machine learning models for PCOS prediction. Another work in this domain had been proposed by Agrawal et al. [31], where the top 30 features from the data were determined using the Chi-square technique, and the underlying state of PCOS was predicted using Random Forest, SVM, Logistic Regression, Gaussian Naive Bayes, and K Neighbors utilizing this reduced feature vector. Moreover, seven types of classifiers were used in the diagnostic model that Hdaib et al. [32] proposed using MATLAB to detect PCOS, and the findings showed that the Linear Discriminant classifier performs the best. In another work, proposed by Reka et al. [33] the follicular fluid sample from 100 women had been extracted and the obtained data set is then preprocessed using Raman spectra and effective feature selection techniques to be utilized for machine learning classification; which were classified using Random Forest, Multilayer Perceptron, Ada Boost and decision tree classification models for detecting PCOS. Again, Boomidevi et al. [34] suggested an artificial Neural network (ANN) model for detecting PCOS at an early stage where a comparative performance analysis had been conducted using different neural network optimizer to explore the best performing ANN design for classifying dataset into two classes: PCOS and Non-PCOS. Another related work in this field has been conducted by Prapty et al. [35], in which they investigated four different machine learning classifiers to categorize PCOS and non-PCOS records and compared their results where the Random Forest classifier outperformed the others; and then employing that Random Forest classifier a decision tree is developed to identify the top features responsible for PCOS. Denny et al. [36] also proposed a framework named ‘i-Hope’ as a paradigm for early identification and prediction of PCOS based on optimum yet promising indicators; here they used a patient survey of 541 records to design the proposed framework, in which 8 potential features from diagnostic and metabolic test results were selected using SPSS and the Principal Component Analysis (PCA) method based on their importance, and then applied to seven types of traditional ML classifiers to find the best performing model.

Since PCOS is associated with a wide range of symptoms as features, a few studies have emphasized on employing various feature reduction approaches before using machine learning models to accelerate the training process. For example, Inan et al. [37] suggested a strong sampling technique that includes both oversampling and undersampling procedures to boost minority samples; then applied two types of feature selection techniques: Chi- Square test for categorical and Analysis of Variance (ANOVA) test for numerical attribute selection; and then applied six types of machine learning classifiers where XGBoost classification model outperformed others. In another relevant article in this domain, Nandipati et al. [38] used RFE-LR, RFI-ECT, SelectKBest/Chi2 and Forward Backward propagation techniques to find the top 10 and 24 features from all 42 features in the dataset, and then applied seven types of traditional ML classifiers in two different types of implementation platforms: Python-Scikit Learn package and RapidMiner; in addition, performance comparisons between various classifiers were assessed utilizing complete (40 features) and selected features (10 and 24 features) to find the best performing classifier. Munjal et al. [39] used a genetic algorithm and WEKA (Waikato Environment for Knowledge Analysis) software to identify the nine primary features associated in (PCOS) illness development and then utilized those reduced set of features over three types of ML classifiers in PyCaret platform to predict the disease using minimal attributes. While, for extracting the most significant attributes from a dataset comprising 26 attributes of 303 instances, Meena et al. [40] suggested an approach based on Neural Fuzzy Rough Set (NFRS) and Artificial Neural Network (ANN) techniques; and then applied those reduced set of features in four different types of classification models to detect PCOS where the performances enhanced in comparison to other five types of traditional feature selection methods.

Now, a potential state-of-the-art approach for several machine learning challenges is ensemble methodologies, as they can significantly improve the performance of a single model's forecasting by training multiple models and combining those results [41]. Recently, a few scholars have used ensemble machine learning methods to generate accurate predictions in various healthcare domains. For example, Jabbar et al. [42] presented an ensemble learning approach to address the problem of categorizing breast cancer data.; Suha et al. [14] applied a hybrid model with CNN and stacked ensemble technique to classify PCOS ultrasound images; Kaushik et al. [43] developed an ensemble of multi-headed ML architectures to forecast the average weekly expenditures on two pain drugs taken by patients etc. A few studies have been found where ensemble techniques had been employed in PCOS identification, for example Gupta et al. [44] applied four types of Boosting ensemble techniques (Adaptive Boost, Gradient Boost, XGBoost and CatBoost) without applying any feature engineering techniques to classify PCOS. Again, Bharati et al. [45] applied hard and soft voting ensemble classifier employing ExtraTree, Random Forest, Gaussian Naive Bayes, LightGBM and eXtreme Gradien Boosting models with reduced set of features selected via recursive feature elimination and univariate feature selection techniques. However, From the prior studies, it can be demonstrated that even though many academics from around the world have suggested contributions where various machine learning strategies have been used to diagnose PCOS; seldom has a researcher looked into the viability and effectiveness of using several ensemble machine learning approaches (bagging, boosting, and stacking) in this circumstance.

From the previous related works it is also observable that, most of the studies have picked a specific reduced subset of features from the existing dataset through applying feature reduction techniques and then performed machine learning classification using that reduced feature subset. But, hardly any studies have explored multiple reduced feature subsets with different numbers and combinations of attributes from the complete dataset. Also, rarely they have investigated how the performances of various machine learning classification techniques might alter when different feature subsets with various combinations and numbers of attributes extracted from multiple feature reduction methods are being used. Furthermore, less attention has been made on investigating and validating whether the retrieved decreased features are genuinely important or not in terms of real-life clinical diagnosis of PCOS via a cross-check involving relevant healthcare specialists.

Thus, this research focuses on addressing these research gaps in this area with the goal of detecting PCOS more effectively and efficiently utilizing the optimum numbers of features. Therefore, a stacking ensemble classifier has been designed, trained, and evaluated as well as the performances of various forms of ensemble and conventional machine learning approaches have been investigated in this study, employing different sets of attributes acquired from feature selection methods.

## 3 Materials and methods

**Figure 1:**
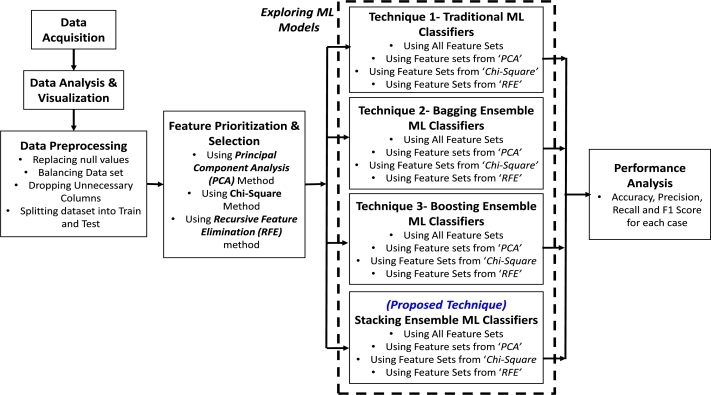
Framework of research methodology.

An extended ensemble ML classifier has been proposed, trained and tested using patient's most significant set of symptom data to differentiate between PCOS & non-PCOS patients in this study. A framework of the research methodology is presented in Fig. 1, which has been thoroughly explained in the following subsections. The research has been conducted through several phases to investigate and prioritize the optimum collection of features essential for predicting PCOS as well as to discover the best performing classification model for PCOS detection using those features. The classification models' inputs are a collection of patient's symptom attributes, and the outputs would be binary responses indicating whether a patient has PCOS or not. After retrieving the data from the repository, the dataset has been analyzed using various visualization approaches to gain a detailed understanding of it and then, it has been meticulously pre-processed to transform it into a clean and suitable dataset that can be used for machine learning. Following that, several sets of reduced features with varied numbers of most significant attributes have been extracted using three different feature selection strategies. Then, for exploring different machine learning techniques several traditional as well as ensemble ML models have been trained and tested employing various sets of features and also a stacked ensemble model has been proposed. A comparative study of several classification models and feature prioritizing techniques has also been performed through different performance matrices to evaluate the efficacy of the classifiers. The methodological procedures that have been used in this research are detailed in the following subsections.

### 3.1 Data acquisition, analysis and visualization

A dataset containing the symptoms along with the PCOS diagnosis findings of patients has been utilized here as the training data for supervised machine learning models, for which a publicly available data collection of PCOS patients from ‘Kaggle’ [46] has been selected. The dataset has been thoroughly investigated for better understanding before employing them for training purpose. The primary analysis of the PCOS records shows that, the dataset comprises a total of 541 records of female patient's data with 45 columns containing various types of clinical information related to PCOS anomaly. One of the columns named ‘PCOS(Y/N)’ has a PCOS diagnosis outcome with ‘Yes’ and ‘No’ values indicating whether or not the patient has PCOS. This feature column has been considered as the target column for the training in this study. When the values of this column are counted, it has been observed that there is an uneven distribution of positive and negative outcomes, as there are 364 entries with ‘No’ indicating ‘NO PCOS’ and 177 entries with ‘Yes’ indicating ‘PCOS’.

**Figure 2:**
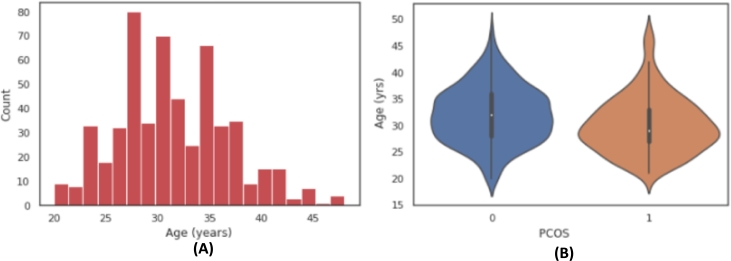
(a) Distribution of Age in Dataset; (b) Relationship of ‘Age’ attribute with target column.

**Figure 3:**
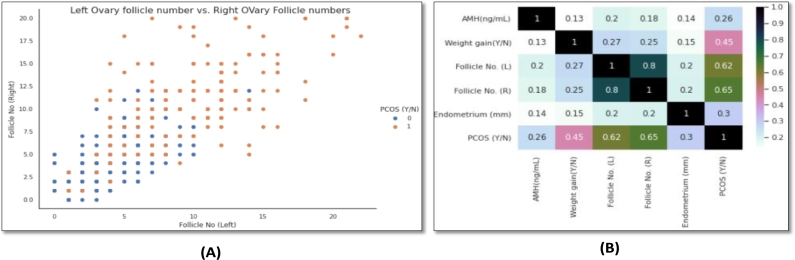
(A) Scatter plot of Left ovary vs Right ovary Follicle numbers with respect to target attribute; (B) Correlation heatmap of some attributes.

Again, the relationship between target column with other attributes has been examined using various visualization approaches. For example, the Fig. 2(A) shows the age distribution of the patient records in the dataset which depicts that the records comprise information on women aged 20 to 50 years old and the Fig. 2(B) shows a violin plot of ‘PCOS(Y/N) vs. Age (yrs)’ which depicts the age range of women with and without PCOS in the dataset. Another example of data visualization has been illustrated in Fig. 3 (A) where the number of follicles in the left ovary vs. right ovary in relation to the goal attribute ‘PCOS(Y/N)’ has been plotted, demonstrating that a larger number of follicles in both the left and right ovary yields the most positive PCOS outcomes. Furthermore, correlation study between the different attributes has been performed using a correlation heatmap to statistically analyze the strength of the relationship between the features, as an example illustrated in Fig. 3 (B). The correlation value ranges from 0 to 1, where with a greater correlation value indicating that the features are highly correlated to each other.

### 3.2 Data preprocessing

In this work, the dataset has been critically analyzed and preprocessed before using them into machine learning models to address the flaws and irregularities in the datasets such as missing or contradictory data samples, inconsistencies, noise, and other issues. The following steps have been employed for preprocessing the dataset.

Firstly, for data preprocessing the null values have been handled. Features with too many null or missing values have been completely removed from the dataset because they don't give any useful information; for example, the feature ‘Unnamed’ in the dataset contains 539 null values for which it has been eliminated. The features comprising a few null values have been substituted with other relevant values; for example, ‘Marriage Status (Yrs)’ and ‘Fast Food (Y/N)’ contains a few null values which have been replaced with 0.

Secondly, the data balancing has been done for making the classes equally distributed for training, as the dataset has imbalanced target attribute with 364 records of non-PCOS and 177 records of PCOS patients. Therefore, the dataset has been over-sampled using ‘Synthetic Minority Oversampling Technique (SMOTE)’ method that generates a synthetic sample of a minority class to eliminate the imbalance in the target attribute values [47]. The mathematical formula followed for SMOTE method has been shown in Equation (1), where $x_{\mathrm{sample}}$ is the sample generated from minority class value *x* and $x_{\mathrm{random}}$ is a randomly chosen value among the nearest neighbors of x with 0≤η≤1. As a result of SMOTE, the dataset instances here have increased to 728 records, including 364 positive and negative PCOS diagnostic results.

(1) $$x_{\mathrm{sample}}=x+\eta (x_{\mathrm{random}}-x)$$

The third step has been to Drop Unnecessary Columns. At this step, superfluous or duplicated columns have been removed in order to improve forecasting accuracy. One of two columns giving the same information has been kept, while the other has been deleted from the dataset. For example, ‘I beta-HCG(mIU/mL)’ numerical column and ‘II beta-HCG(mIU/mL)’ categorical attribute provide same information from which ‘II beta-HCG(mIU/mL)’ has been discarded. Also the unnecessary columns ‘Sl. No.’ and ‘Patient File No.’ have been discarded from the dataset as they contain simply the serial numbers, patient's file no which can be ignored for further analysis.

The next step is data normalization in which the values of the dataset are normalized using the MinMax Scalar approach to reduce the influence of variance in measurement units of different features and eliminate attribute bias with sensitivities [48]. The MinMax scaller follows the Equation (2) for rescaling the values of the feature range between 0 to 1. In the Equation (2), $x_{\mathrm{scaled}}$ is the rescaled value generated from the original vale *x* where $x_{min}$ and $x_{max}$ are the minimum and maximum values of that attribute $A_{i}$.

(2) $$x_{\mathrm{scaled}}=(x-x_{min})÷(x_{max}-x_{min})$$

Finally, as the last step of data preprocessing, the dataset has been divided into train and test datasets for applying them to classification models of machine learning, with 30% of the instances randomly assigned to the test dataset and the remaining 70% assigned to the train dataset.

### 3.3 Feature selection

Feature selection is an efficient method for picking the most significant attributes and avoiding unimportant features to improve the prediction capacity and accuracy of machine learning algorithms [49]. It is the process of exploring the best subset(s) of features to assure the finest potential data description. In this study, the dataset contains 40 attributes after preprocessing, which may lower the accuracy of the classifier if all of the less significant ones are taken into account. Thus, the features in this context have been prioritized and selected rigorously using three types of feature selection techniques to find out the optimal set of features from the PCOS data set. The techniques have been described hereafter:

• **Chi-Square Technique:** Chi-square feature selection technique is one of the most frequent and helpful feature selection strategies used in machine learning [50]. It conducts a numerical test that calculates deviation from the anticipated distribution when the feature event is independent to the class value and prioritizes features by examining the relationship between them [51]. The formula for the chi-square feature selection has been shown in Equation (3). In the equation, the real number of observations in the dataset that fit into a particular feature i are the observed values and the number of observations which are anticipated to occur is represented by the expected values. Here, the prioritized features are chosen according to the best scores of $\chi^{2}$. In case of implementation, the python ‘SelectKBest’ function has been utilized, which implemented the chi-square numeric test with k=n, where k is the number of features that will be selected by the algorithm and then picked n features from the dataset's 40 features based on the highest scores.
  (3) $$\chi^{2}=\sum_{i=1}^{n}\frac{{\left({\text{Observed Value}}_{i}-{\text{ExpectedValue}}_{i}\right)}^{2}}{{\text{ExpectedValue}}_{i}}$$
• **Principal component analysis (PCA) Technique:** The second type of technique that has been used for feature selection in this study is the Principal component analysis (PCA) method, which is an efficient dimension reduction tool for feature prioritization utilizing numerical analysis which is accomplished by assessing the correlation between characteristics in order to determine the most important or principal components [52], [53]. PCA maps and reconstructs the original n-dimensional features to the required k-dimensional features (k<n), where the k-dimensional features are new orthogonal attributes termed as principle components that minimize data redundancy to accomplish the dimension reduction goal [54]. In this scenario, the python ‘PCA’ function from Scikit-learn has been utilized with the PCA variance, to determine the most important n features.
• **Recursive Feature Elimination (RFE) Technique:** Recursive Feature Elimination, or RFE, is an efficient wrapper-type strategy that has been utilized in this study for removing features from a training sample for feature selection which ranks the set of attributes and eliminates them at the bottom that contribute the lowest to the categorization [55]. This approach is basically a recursive process that employs several machine learning techniques at its foundation, wrapped in the RFE methodology, and therefore feature importances are calculated at each iteration, with the least relevant one being eliminated to pick the prioritized features [56], [57]. The RFE function from the RFE class provided by the scikit-learn Python machine learning library has been employed here for implementation

To explore the highly significant attributes that would yield the best performing accuracy when used in machine learning models, each type of feature selection approach selects the top 35, 30, 25, and 20 features from the PCOS dataset of 40 features. The algorithm followed for extracting the reduced set of features from the dataset in this study is shown in Algorithm 1. Then employing those different sets of features the machine learning classifiers are trained, tested and evaluated through different performance metrics.

**Algorithm 1:**
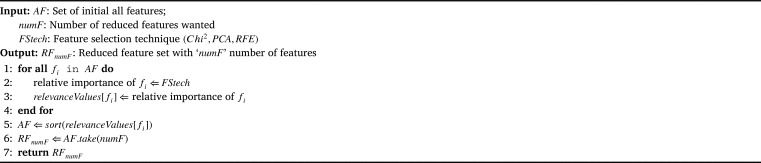
Pseudo Code for Feature Selection.

### 3.4 Machine learning models

Classification is a machine learning technique that uses a model learned from training data to forecast the category of samples and therefore maps or classifies data instances into the associated class labels which have been predefined in the provided dataset [58]. In this study, for training the machine learning models with an aim to categorize between PCOS & non-PCOS classes from their symptom data, four types of techniques have been employed (see Fig. 1). The predictive models have been trained, tested and evaluated using different sets of features from the dataset. The machine learning techniques have been discussed briefly below.

#### 3.4.1 Existing machine learning techniques

• Traditional ML Classifiers: Although for conducting predictive analytics, a number of classification strategies with the ability to predict outcomes are employed, certain traditional machine learning classification methods have been widely employed to estimate a variety of clinical anomalies in numerous research. Here, technique 1 employs five kinds of well-known and widely utilized traditional machine learning classification techniques with fundamental algorithmic structure which are appropriate to this target area. The models are Logistic Regression classifier, Support Vector Machine classifier, Decision Tree classifier, K-Nearest Neighbour classifier and Naive Bayes classifier. These machine learning classifiers have been applied extensively in a variety of healthcare-related predictive studies. Table 1 shows a summary of these traditional machine learning models used in various clinical prediction related studies.

**Table 1:** A summary of traditional machine learning classifiers used for different healthcare predictive studies.

Classifiers	Brief Description	Examples of healthcare predictions	References
Logistic Regression	A probabilistic-based statistical model in which the classifier assesses the association between the dependent variable as target class and independent variables or features for a given dataset using a logistic function [59]	Chronic disease prediction, ovarian cancer classification, Alzheimer's disease detection etc.	Nusinovici et al. [60], Octaviani et al. [61], Xiao et al. [62]

Support Vector Machine	A hyperplane is chosen, which is a line that can discover the coefficients, separate samples in the variable space with the best detachment of the classes [63]	PCOS detection, heart disease diagnosis, cervical cancer detection etc.	Sengur et al. [64], Bharati et al. [65], Zhang et al. [66]

Decision Tree	Estimates entropy and information gain for each attribute over a provided training sample and analyzes each feature at each node of a top-down tree for classification [67]	Parkinson's disease identification, COVID-19 diagnosis, coronary artery disease diagnosis etc.	Syapariyah et al. [68], Yoo et al. [69], Ghiasi et al. [70]

K-Nearest Neighbour	It's a instance-based learning that considers local approximation presuming that similar data are close together & computation is conducted until classification [71]	Diabetes detection, chronic kidney disease prediction, Ovarian cancer classification etc.	Suyanto et al. [72], Devika et al. [73], Alqudah et al. [74]

Naive Bayes	A fundamental probabilistic based classification strategy for predicting class membership probability by computing the likelihood of membership for each category [75]	Breast cancer detection, brain tumor detection, thyroid detection etc.	Kharya et al. [76], Zaw et al. [77], Chandel et al. [78]
• Bagging Ensemble ML classifiers:
  A bagging classifier or bootstrap aggregation classifier is an ensemble method that fits multiple base classification models on randomized subsets of the dataset with the same weights given to each model and then aggregates their individual predictions to generate a final result [99]. In this study, Random Forest classifier has been used for classification as a type of bagging classifier which is created based on the aggregation of numerous decision tree base classifiers. During the evolution of a decision tree, Random Forest employs random subset or feature projection which means rather than using all of the parameters in one tree, each decision tree in Random Forest selects only a subset of variables at every prospective splits [100]. A number of researchers have used random forest classifier successfully to the various domains of healthcare predictive analysis. A brief summary in this regard has been shown in Table 2.

**Table 2:** A summary of bagging and boosting ensemble machine learning classifiers used for different healthcare predictive studies.

Type	ML Classifiers	Brief Description	Examples of healthcare predictions	References
Bagging ensemble	Random Forest classifier	Integrates bootstrap aggregation (bagging) and random feature selection to create a set of decision trees with controlled variation that can anticipate the corresponding output activity class [79]	PCOS detection, lymph disease diagnosis, thyroid disorder analysis etc.	Tiwari et al. [80], Azar et al. [81], Mishra et al. [82]

Boosting ensemble	Gradient Boosting classifier	It is an ensemble forward learning model which eliminates all weaker predictors in favor of a stronger one using an upgraded version of the decision tree, in which each successor is selected using the refined structure score, gain computation, and advanced approximations [83]	Lung cancer detection, diabetes diagnosis, Leukemia prediction etc.	Chandrasekar et al. [84], Bahad et al. [85], Deif et al. [86]
	eXtreme Gradient (XG) Boosting	This approach is scalable and efficient form of gradient boosting that improves on two fronts: tree construction speed and a novel distributed algorithm for tree searches [87]	Heart disease detection, chronic kidney disease diagnosis, breast cancer detection etc.	Ashish et al. [88], Ogunleye et al. [89], Inan et al. [90]
	Adaptive Boosting classifier	It's an adaptive classifier that leverages the results of various weak learning algorithms to substantially enhance performance and provide an effective predictor for the boosted classifier's final output [91]	Endometrial cancer prediction, Hepatitis disease detection, cancer classification etc.	Wang et al. [92], Akbar et al. [93], Lu et al. [94]
	Categorical Gradient (CAT) Boosting	It is an implementation of Gradient Boost classifier that employs ordered boosting with categorical features and uses binary decision trees as underlying predictors [95]	Parkinson's disease prediction, COVID-19 detection from blood samples, diabetes risk prediction etc.	Al et al. [96], Abayomi et al. [97], Kumar et al. [98]
• Boosting Ensemble ML classifiers:
  Boosting is an ensemble machine learning approach in which a random sample data is chosen, fitted with a model, and then trained in a sequential manner, combining a set of weak learners into a strong learner with an aim to minimize training errors, with every model attempting to compensate for the shortcomings of the previous model [101]. Based on the different ways of producing and aggregating weak learners during the sequential approach, boosting algorithms can be categorized into different types. In this study, four types of widely utilized variations of boosting ensemble technique have been employed which are: Gradient (Grad) Boosting classifier, Adaptive (Ada) Boosting Classifier, eXtreme Gradient (XG) Boosting classifier and CAT Boosting classifier. These classifiers have been considered here because they have been successfully applied to a range of challenges in the field of healthcare predictive modeling, as a brief summary shown in Table 2.

#### 3.4.2 Proposed machine learning classifiers

**Figure 4:**
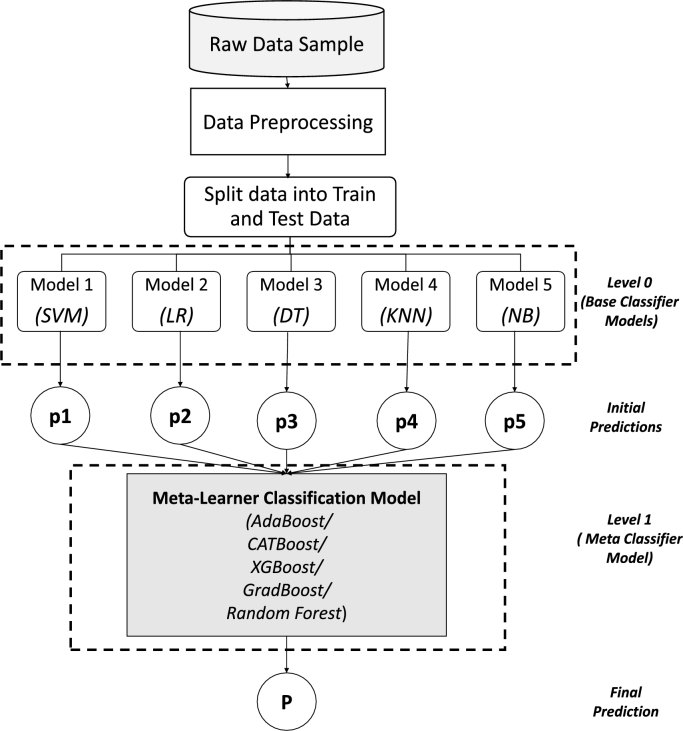
Basic Framework of the Proposed Stacking Ensemble Technique.

To achieve greater forecasting performance than a single classifier, ensemble learning employs multiple classifiers; where Stacking ensemble learning is the technique that use a meta-classifier to aggregate various weak classifiers. The likelihood of belonging to a class is returned by the first layer's classifiers as a meta-feature; than these meta-features with the dataset are the input for the meta-classifier in the second level. Finally, the classifier's output can be either 1 or 0 [102]. A stacking ensemble based ML classification approach has been proposed for predicting the PCOS or non-PCOS criteria in this study that differs from bagging and boosting approaches in the following perspectives: (a) it evaluates diverse weak classifiers and simultaneously trains them.; (b) then aggregates them by training a meta-learner to generate a forecast relying upon every weak learner's individualized predictions; and (c) hence, it reduces variance and improves the learning process' predictive power. [103]. The basic framework of the proposed stacking ensemble machine learning technique has been illustrated in Fig. 4.

The proposed model is a multi-level stacked ensemble model where after preprocessing the raw data sample, it is being divided into train and test data and then initially sent to the base learners of level 0. At this phase, the five types of widely utilized traditional machine learning classifiers have been considered to be the weak learners or base classifiers at level 0 of the stacked model, which are: Logistic Regression (LR), Support Vector Machine (SVM), Decision Tree (DT), K-Nearest Neighbour (KNN) and Gaussian Naive Bayes (NB) classifiers (see Table 1). These base models are each independently trained by employing their own prediction models, producing forecasts denoted by the letters p1, p2, p3, p4, and p5 in Fig. 4. The level 1 models are therefore given the level 0 forecasts, and a single classification algorithm, or meta-learner, learns to produce the final prediction from all of these. At level 1, the meta-learner is built using a stronger machine learning classifier. Five different classifier types have been investigated here as meta learners at level 1 while maintaining the same basis models at level 0, which has produced five different versions of the suggested model in an effort to find the one performing most effectively. The meta learner is one of the five types of bagging or boosting classifiers described previously in Table 2, which is eventually trained on top of level 0 to provide the final output depending on the forecasts provided by the base models. The output of the level 0 classification models serves as the input for the level 1 algorithms rather than features of the raw data. As a result, a stacked ensemble machine learning classifier has been suggested that incorporates five different classic classifiers as base models with one boosting or bagging type of classifier as meta learner, in order to distinguish between patients with PCOS and those who do not have PCOS.

## 4 Results and findings

In this study, to evaluate and compare the efficacy of the predictive models for PCOS detection, total four types of ML techniques have been performed employing fifteen varieties of ML classifiers including the traditional (five models), bagging (one model), boosting (four models) and proposed (five models) techniques. All the experiments have been simulated using patient symptom dataset for classifying the records into PCOS and non-PCOS criteria. Furthermore, to explore the optimum and most significant attributes from the dataset, three types of feature selection methods (Chi-square, PCA and RFE) have been employed which picked the top 35, 30, 25 and 20 features out of the 40 features of the dataset. Each ML model's performance has then been evaluated employing these different sets of features acquired from feature selection techniques.

The performance of different varieties of machine learning algorithms utilizing different sorts of feature sets is analyzed using four performance measures, which are Accuracy, Precision, Sensitivity (recall), and F1 score, to investigate the efficacy of the prediction analysis [104]. The performance metrics are primarily based on a comparison of anticipated and actual values that investigates number of correct and incorrect predictions from the training sample, which is divided into four categories: True Positive (TP) that is both the true and predicted values are positive; True Negative (TN) in which both the original and the anticipated values are negative.; False Positive (FP) a where the actual value is negative but the anticipated result is positive and lastly False Negative (FN) where the actual value is positive, but the predicted result is negative. Based on these evaluations, the performance measures utilized here can be stated as Equations (4), (5), (6), and (7):

(4) Accuracy=(TP+TN)÷(TP+TN+FP+FN)

(5) Precision=TP÷(TP+FP)

(6) Recall(Sensitivity)=TP÷(TP+FN)

(7) F1−score=2⁎(Precision⁎Sensitivity)÷(Precision+Sensitivity)

The findings of this rigorous evaluation process have been shown in Table 3, Table 4, Table 5, Table 6; where Table 3 shows the accuracy, Table 4 shows the precision, Table 5 shows the recall and Table 6 shows the F1-score of different models using different sets of features. The best performance results from each column have been highlighted in the tables.

**Table 3:** Accuracy Comparison of ML models using different set of features.

Type	Classification Models	40 Feat.	35 Feat. Accuracy			30 Feat. Accuracy			25 Feat. Accuracy			20 Feat. Accuracy		
			Chi^2^	PCA	RFE	Chi^2^	PCA	RFE	Chi^2^	PCA	RFE	Chi^2^	PCA	RFE
Trad Tech	SVM	0.507	0.89	0.511	0.534	0.871	0.911	0.893	0.708	0.921	0.679	0.609	0.583	0.611
	Log. Reg	0.872	0.89	0.886	0.895	0.92	0.911	0.893	0.702	0.921	0.688	0.714	0.611	0.711
	DecisionTree	0.836	0.822	0.868	0.863	0.804	0.78	0.879	0.716	0.864	0.713	0.653	0.664	0.65
	KNN	0.685	0.816	0.667	0.685	0.816	0.864	0.813	0.622	0.879	0.736	0.696	0.716	0.707
	NaiveBayes	0.868	0.84	0.863	0.863	0.859	0.766	0.864	0.74	0.808	0.76	0.565	0.585	0.564

Bag. Tech	RandomForest	0.889	0.89	0.906	0.902	0.89	0.907	0.902	0.851	0.916	0.86	0.708	0.793	0.8

Boosting Tech	GradBoosting	0.872	0.877	0.883	0.893	0.89	0.888	0.93	0.853	0.874	0.832	0.777	0.715	0.706
	XG Boosting	0.89	0.877	0.897	0.864	0.881	0.907	0.897	0.853	0.85	0.864	0.689	0.675	0.715
	AdaBoosting	0.886	0.896	0.869	0.883	0.853	0.902	0.902	0.871	0.864	0.869	0.756	0.8	0.686
	CATBoosting	0.9	0.863	0.841	0.832	0.9	0.916	0.916	0.865	0.879	0.85	0.789	0.799	0.725

Proposed Tech (stacking)	Meta learner-**Grad Boost**	**0.927**	0.922	**0.932**	0.918	0.926	**0.953**	**0.943**	0.883		0.911	0.853	**0.832**	0.86
	Meta learner-XGBoost	0.913	0.918	0.918	0.922	0.926	0.935	0.924	0.859	0.942	0.893	**0.871**	0.822	**0.897**
	Meta learner-AdaBoost	0.922	**0.934**	0.927	**0.936**	**0.946**	0.931	0.938	0.883	0.942	0.907	0.808	0.802	0.893
	Meta learner-CATBoost	0.913	0.89	**0.932**	0.918	0.933	0.943	0.933	**0.908**	0.947	0.893	0.802	0.825	0.802
	Meta learner-RandForest	0.909	0.883	0.913	0.927	0.924	0.918	0.925	0.89	0.925	**0.916**	0.802	0.812	0.807

**Table 4:** Precision Comparison of ML models using different set of features.

Type	Classification Models	40 Feat.	35 Feat. Precision			30 Feat. Precision			25 Feat. Precision			20 Feat. Precision		
			Chi^2^	PCA	RFE	Chi^2^	PCA	RFE	Chi^2^	**PCA**	RFE	Chi^2^	PCA	RFE
Trad Tech	SVM	0.65	0.874	0.514	0.541	0.855	0.912	0.894	0.799	0.921	0.68	0.61	0.582	0.613
	Log. Reg	0.872	0.878	0.887	0.897	0.91	0.912	0.893	0.791	0.921	0.69	0.715	0.621	0.711
	DecisionTree	0.837	0.802	0.868	0.863	0.783	0.785	0.879	0.715	0.865	0.715	0.654	0.66	0.651
	KNN	0.697	0.798	0.667	0.685	0.795	0.865	0.816	0.602	0.882	0.736	0.698	0.715	0.71
	NaiveBayes	0.874	0.841	0.869	0.865	0.873	0.805	0.865	0.741	0.829	0.76	0.565	0.586	0.565

Bag. Tech	RandomForest	0.888	0.878	0.907	0.902	0.878	0.912	0.902	0.85	0.917	0.861	0.708	0.792	0.802

	GradBoosting	0.873	0.866	0.885	0.894	0.88	0.888	0.93	0.847	0.876	0.836	0.775	0.716	0.706
Boosting Tech	XG Boosting	0.891	0.879	0.898	0.865	0.882	0.909	0.897	0.886	0.852	0.865	0.688	0.67	0.715
	AdaBoosting	0.886	0.886	0.87	0.883	0.837	0.902	0.902	0.864	0.866	0.869	0.755	0.822	0.67
	CATBoosting	0.901	0.872	0.843	0.836	0.89	0.92	0.917	0.85	0.88	0.854	0.78	0.796	0.724

Proposed Tech (stacking)	Meta learner **Grad Boost**	**0.927**	0.921	0.931	0.918	0.925	**0.952**	**0.945**	0.868		0.912	0.85	**0.835**	0.862
	Meta learner XGBoost	0.914	0.895	0.918	0.923	0.922	0.934	0.925	0.853	0.942	0.893	**0.867**	0.823	**0.895**
	Meta learner AdaBoost	0.922	**0.925**	0.927	**0.936**	**0.948**	0.942	0.937	0.872	0.931	0.908	0.81	0.802	0.893
	Meta learner CATBoost	0.914	0.864	**0.932**	0.918	0.935	0.944	0.933	**0.897**	0.947	0.893	0.801	0.826	0.806
	Meta learner RandForest	0.909	0.868	0.914	0.927	0.925	0.919	0.924	0.876	0.926	**0.915**	0.802	0.811	0.807

**Table 5:** Recall Comparison of ML models using different set of features.

Type	Classification Models	40 Feat.	35 Feat. Recall			30 Feat. Recall			25 Feat. Recall			20 Feat. Recall		
			Chi^2^	PCA	RFE	Chi^2^	PCA	RFE	Chi^2^	**PCA**	RFE	Chi^2^	PCA	RFE
	SVM	0.513	0.886	0.506	0.531	0.864	0.911	0.893	0.79	0.922	0.67	0.612	0.583	0.622
Trad	Log. Reg	0.872	0.878	0.886	0.896	0.914	0.911	0.893	0.79	0.921	0.68	0.725	0.62	0.71
Tech	DecisionTree	0.835	0.805	0.867	0.863	0.778	0.78	0.879	0.8	0.867	0.73	0.655	0.66	0.655
	KNN	0.687	0.822	0.667	0.685	0.809	0.864	0.813	0.61	0.887	0.74	0.698	0.725	0.711
	NaiveBayes	0.868	0.798	0.864	0.863	0.812	0.766	0.864	0.78	0.815	0.86	0.56	0.596	0.565

Bag. Tech	RandomForest	0.888	0.878	0.906	0.902	0.878	0.907	0.902	0.85	0.922	0.86	0.709	0.793	0.803

Boosting Tech	GradBoosting	0.873	0.86	0.883	0.893	0.873	0.888	0.93	0.82	0.871	0.83	0.777	0.717	0.71
	XG Boosting	0.89	0.878	0.897	0.864	0.881	0.907	0.897	0.79	0.853	0.86	0.689	0.673	0.712
	AdaBoosting	0.889	0.882	0.869	0.883	0.837	0.902	0.902	0.85	0.862	0.88	0.754	0.823	0.671
	CATBoosting	0.9	0.869	0.841	0.832	0.877	0.916	0.916	0.85	0.886	0.85	0.781	0.794	0.724

Proposed Tech (stacking)	Meta learner **Grad Boost**	**0.927**	0.92	0.932	0.918	0.929	**0.952**	**0.943**	0.87		0.91	0.851	0.822	0.860
	Meta learner XGBoost	0.914	0.904	0.918	0.923	0.921	0.934	0.924	0.83	0.942	0.89	**0.877**	0.826	**0.894**
	Meta learner AdaBoost	0.922	**0.925**	0.927	**0.936**	**0.948**	0.942	0.938	0.87	0.934	0.91	0.812	0.805	0.893
	Meta learner CATBoost	0.914	0.864	**0.933**	0.918	0.934	0.944	0.933	**0.91**	0.944	0.89	0.804	**0.827**	0.807
	Meta learner Rand Forest	0.909	0.877	0.914	0.927	0.925	0.919	0.923	0.88	0.922	**0.920**	0.802	0.815	0.81

**Table 6:** F1-Score Comparison of ML models using different set of features.

Type	Classification Models	40 Feat.	35 Feat. F1-Score			30 Feat. F1-Score			25 Feat. F1-Score			20 Feat. F1-Score		
			Chi^2^	PCA	RFE	Chi^2^	PCA	RFE	Chi^2^	**PCA**	RFE	Chi^2^	PCA	RFE
Trad Tech	SVM	0.367	0.88	0.427	0.5	0.864	0.911	0.893	0.79	0.922	0.69	0.691	0.674	0.683
	Log Reg	0.872	0.878	0.886	0.895	0.914	0.911	0.893	0.79	0.921	0.68	0.712	0.678	0.711
	DecisionTree	0.835	0.804	0.868	0.863	0.778	0.78	0.879	0.79	0.866	0.71	0.623	0.62	0.664
	KNN	0.681	0.805	0.667	0.685	0.809	0.864	0.813	0.61	0.878	0.74	0.684	0.786	0.716
	Naive Bayes	0.867	0.812	0.863	0.863	0.812	0.766	0.864	0.71	0.814	0.86	0.543	0.555	0.585

Bag. Tech	RandomForest	0.889	0.878	0.912	0.902	0.878	0.907	0.902	0.85	0.91	0.86	0.781	0.685	0.853

Boosting Tech	GradBoosting	0.872	0.863	0.883	0.892	0.873	0.888	0.93	0.83	0.870	0.81	0.793	0.769	0.731
	XG Boosting	0.89	0.878	0.897	0.864	0.881	0.907	0.897	0.82	0.851	0.86	0.683	0.641	0.72
	AdaBoosting	0.887	0.0884	0.869	0.883	0.837	0.902	0.902	0.85	0.863	0.87	0.783	0.864	0.602
	CATBoosting	0.9	0.87	0.841	0.831	0.877	0.916	0.916	0.85	0.877	0.85	0.785	0.769	0.725

Proposed Tech (stacking)	Meta learner **Grad Boost**	**0.927**	0.92	0.932	0.918	0.929	**0.950**	**0.943**	0.87		0.91	**0.891**	**0.885**	0.82
	Meta learner XGBoost	0.913	0.899	0.918	0.922	0.921	0.934	0.924	0.84	0.923	0.89	0.882	0.869	0.869
	Meta learner AdaBoost	0.922	**0.925**	0.927	**0.936**	**0.948**	0.942	0.938	0.87	0.933	0.91	0.814	0.801	**0.881**
	Meta learner CATBoost	0.913	0.864	**0.932**	0.918	0.934	0.944	0.933	**0.89**	0.943	0.89	0.809	0.824	0.81
	Meta learner Random Forest	0.909	0.872	0.913	0.927	0.925	0.919	0.923	0.87	0.923	**0.921**	0.811	0.829	0.818

### 4.1 Comparative performance analysis of the proposed technique with other ML techniques

Analyzing the evaluation results from Table 3, Table 4, Table 5, Table 6, it can be observed that, the performances of the classifiers enhance significantly using the proposed stacked ensemble techniques. For example, incorporating all the 40 features the best performance has been achieved using the proposed stacked ensemble classifier with Gradient boosting model as meta learner attaining 92.7% accuracy, 92.7% precision, 92.7% recall and 92.7% F1 score. Also it is noticiable that, each of the stacked ensemble models has acquired accuracy performance over 90% using the proposed technique with all features whereas the other models typically have less than or equal to 90% accuracy. Similar findings are also observed in case of using all the reduced set of features (set of 35,30,25 and 20 features) acquired from feature selection techniques, where the five varieties of proposed ML models outperform the other types of models in terms of all the performance metrics.

**Figure 5:**
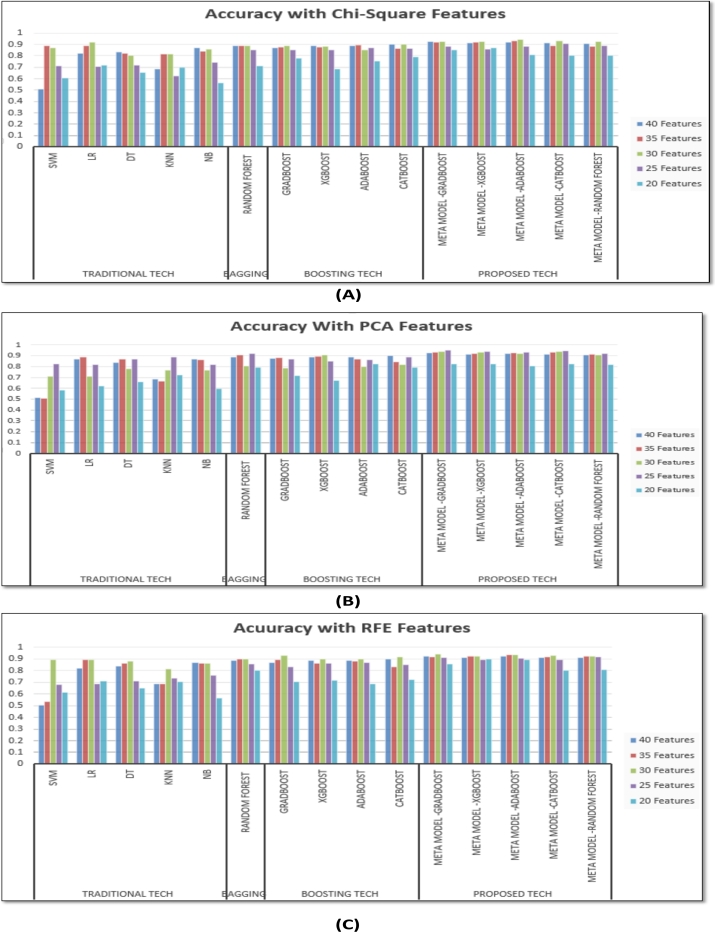
Comparative accuracy analysis of ML models with different sets of (A)Chi-Square, (B)PCA and (C)RFE features.

Fig. 5 graphically illustrates the comparative analysis of the accuracy of different ML models incorporating different feature sets where Fig. 5 (A) shows the accuracy of the models with features selected using chi-square method, Fig. 5 (B) shows accuracy with PCA features and Fig. 5 (C) shows comparative accuracy with RFE features. Each of the graphical representation compares the accuracy performances of four techniques with different classification models employed in this study using 40 features, 35 features, 30 features, 25 features and 20 features selected using chi-square (see 5 (A)), PCA (see 5 (B)) and RFE (see 5 (C)) feature selection method. From the graphical representation it is clearly visible that the performances of the proposed stacking ensemble models are comparatively higher than the other models in case of all types of feature selection methods. Thus, the results acquired from evaluating the ML techniques with different performance metrics clearly indicate that, the proposed stacking ensemble techniques provide a better performance for classifying the dataset into PCOS and non-PCOS classes.

### 4.2 Results of feature selection with different ML techniques

The different feature selection methods utilized here have selected different sets of attributes employing their own methodologies. A list has been given in Table 7 which shows the top 25 features that have been picked using three types of feature selection methods. It is apparent from the Table 7 that the most important attributes of the three techniques are evidently nonidentical. From the table it is observable that, the three set of top 25 features differs from each other such as both PCA and RFE has considered ‘Endrometrium’ (endrometrium thickness of follicles) as a significant feature but Chi-square technique has not selected it; on the other hand chi-square technique has selected ‘Marriage Status (Yrs)’ as an important feature but PCA technique has not prioritized it; and so on. These results indicate that different feature selection techniques pick different combinations of features from the dataset and thus it is necessary to investigate which set of feature provides the best performance.

**Table 7:** Top 25 dominant features prioritized by three types of feature selection methods.

	Chi-Square	PCA	RFE
1	Age (yrs)	Weight (Kg)	Weight (Kg)
2	Weight (Kg)	BMI	Height (Cm)
3	BMI	WeightGain Y/N	BMI
4	Cycle (R/I)	Waist (inch)	Marraige Sta (yr)
5	Cycle length	Hip (inch)	Cycle (R/I)
6	Marraige Sta. (yr)	hair growth-Y/N	Endometrium
7	Pregnant (Y/N)	Follicle No. (L)	Pregnant (Y/N)
8	No. of abortions	Fast food (Y/N)	Pulse rate (bpm)
9	LH (mIU/mL)	Skin dark (Y/N)	FSH (mIU/mL)
10	FSH (mIU/mL)	Follicle No. (R)	LH (mIU/mL)
11	Hip (inch)	Avg. F size (L)	TSH (mIU/L)
12	Waist (inch)	Avg. F size (R)	PRG (ng/mL)
13	AMH (ng/mL)	Cycle (R/I)	No. of abortions
14	Vit D3 (ng/mL)	Pimples (Y/N)	WeightGain
15	PRG (ng/mL)	Hair loss (Y/N)	hair growth-Y/N
16	WeightGain-Y/N	Height (Cm)	Skin dark (Y/N)
17	hair growth-Y/N	AMH (ng/mL)	Hair loss (Y/N)
18	Skin dark (Y/N)	Endometrium	Pimples (Y/N)
19	Hair loss (Y/N)	FSH/LH	Fast food (Y/N)
20	Pimples (Y/N)	Cycle length	Follicle No. (R)
21	Fast food (Y/N)	Hb (g/dl)	Follicle No. (L)
22	Reg.Exer.-Y/N	Vit D3 (ng/mL)	Cycle length
23	Follicle No. (L)	RBS (mg/dl)	Avg. F size (L)
24	Follicle No. (R)	Age (yrs)	Reg.Exer.-Y/N
25	Avg. F size (L)	BP Systolic	RR (breaths/min)

From the comparative evaluation with graphical representation in Fig. 5, another significant finding is that, the accuracy of the models employing Chi-square and RFE feature selection methods gradually enhances when the number of features have been reduced from 40 features to 30 selected features; but then the performances start decreasing for the selected 25 and 20 features for almost all the models. The highest accuracy for most of the models employing chi-square and RFE feature selection method has been acquired with top 30 selected features. Here, the highest accuracy with Chi-square feature selection method has been achieved using stacking ensemble classifier with ‘AdaBoost’ model as meta learner which is 94.6% using top 30 features; and the highest accuracy with RFE feature selection method has been achieved using stacking ensemble classifier with ‘GradBoost’ model as meta learner which is 94.3%.

**Figure 6:**
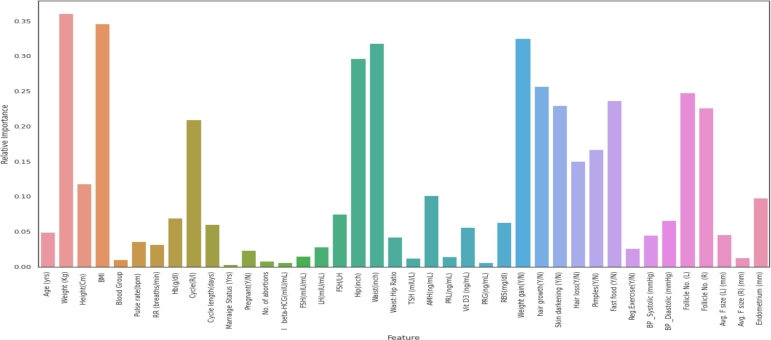
Relative importance of features based on PCA technique.

However, when using the PCA feature selection approach, most of the models' accuracy consistently improves with reduced features and has reached its peak with the top 25 features. Fig. 6 graphically displays the relative importance of all the features of the dataset based on PCA technique. Using the top 25 features selected via the PCA approach, the maximum accuracy being 95.7% has been achieved in this context with a stacking ensemble classifier with the ‘GradBoost’ model as the meta learner. The most significant 25 attributes providing the best performance that has been explored using PCA technique are shown in Table 8. In this table the top selected features are further grouped based on the real-time clinical feature categories under the supervision of three expert clinicians in this relevant field. Furthermore, the identified 25 features of Table 8 have been discussed with three healthcare specialists and according to them, the selected criteria have been rightly regarded to be the crucial predictive attributes in terms of practical PCOS identification. This investigation shows that the PCA technique's minimal yet optimal number of features can not only be used to deliver the best performance with ML classifiers, but can also be effectively utilized to implement a real-time autonomous PCOS detection model in the future.

**Table 8:** Categorization of top 25 dominant features based on PCA technique.

Feature Categories	Features	
**Demographics**	Age (yrs)	

**Vital Signs**	Weight Gain	Cycle (R/I)
	Body hair growth	Pimples
	Skin darkening	Hair loss

**Patient History**	BMI	Waist (inch)
	Cycle length	Hip (inch)
	Weight (Kg)	Height (Cm)
	BP Systolic	

**Laboratory Diagnosis Outcomes**	Follicle No (L)	Follicle No (R)
	Avg. F size (L)	Avg. F size (R)
	Endometrium Thickness	

**Comorbidities**	Hb (g/dl)	AMH (ng/mL)
	FSH/LH	RBS (mg/dl)
	Vit D3 (ng/mL)	

Therefore, from the overall performance analysis, it is observable that, the traditional machine learning models, are explored as being weak classifiers in the context of this dataset and produce the weaker performances which eventually gives a bit better result through bagging and boosting type of ensemble classification models. On the other hand, as a result of the proposed stacked ensemble models' robust formulation, which incorporates the predictive analytics of several classifiers, the results show that each version of the suggested stacked ensemble approaches yields superior outcomes. Also, in terms of feature engineering, the selected features through PCA technique provide better results employing the classification models while chi-square technique provides least performances. Thus, the results of performance analysis indicate that, the machine learning model employing the proposed stacking ensemble method with five classifiers (SVM, LR,DT, KNN,NB) as base models and GradBoost classifier as meta learner; utilizing the top 25 attributes from the dataset selected through PCA feature selection technique has been explored to be the highest performing classification model with 95.7% accuracy, 95.2% precision, 95.2% recall and 95.0% F1-score that outperforms all other models to classify PCOS and non-PCOS criteria.

## 5 Discussion

In this article, three types of ensemble machine learning strategies (bagging, boosting and stacking) with multiple classifiers have been explored, trained and tested along with traditional machine learning techniques to classify PCOS and non-PCOS data. Most of the previous studies in this area were based on traditional ML classifiers. However, recently a few researchers have focused on applying ensemble techniques in PCOS detection, but their exploration techniques are based on typical bagging, boosting or voting type of ensemble models [29], [45]. To the best of our knowledge, the proposed technique based on stacking ensemble classification approach where both traditional as well as boosting or bagging ensemble models are aggregated to provide a stronger prediction is a unique solution in this domain. Here in the stacked ensemble architecture, five types of weak traditional ML classifiers are used as the base models and then their predictions are integrated in a stronger meta-learner classification model to provide the final prediction. One from five types of boosting or bagging classifier has been used as the meta learner in the proposed stacked ensemble model to explore the best performing model where the highest performance has been acquired with 95.7% accuracy which is also higher than previous studies employing identical dataset. For instance, Bharti et al. [45] had acquired the best accuracy of 91.12% with voting ensemble technique, Nandipati et al. [38] showed 93.12% accuracy with Random Forest classifier, Prapty et al. [35] acquired 93.5% accuracy employing Random Forest classifier and so on.

Furthermore, using feature selection strategies, the majority of previous studies randomly picked a specified number of features. For example, Bharti et al. [65] applied ML classifiers with ten statistically significant features based on p-values, Inan et al. [37] proposed to use most significant top twelve features, Danaei et al. [29] had acquired best accuracy employing 28 features selected using Random Forest embedded feature selection technique and so on. However, hardly any study has investigated at how changing the numbers and combinations of features selected using that same feature selection method can affect the prediction result. Therefore, in this study, three distinct types of feature selection techniques (Chi-square, PCA and RFE) have been applied to identify the optimum features that are required for effective forecasting from the dataset's 40 attributes. Each of the feature selection techniques have been used to select different feature sets with top 35,30,25 and 20 attributes. And then the performances of the proposed stacking ensemble models as well as other traditional, bagging and boosting ensemble models are evaluated using those vast varieties of selected feature sets through performance metrics (accuracy, precision, recall and F1-scores). As per the findings of the comparative analysis, it has been observed that, the accuracy of most models using the feature set selected via Chi-square and RFE strategies improves up to the top 30 features and thereafter gradually diminishes, whereas in case of PCA feature selection approach the accuracy enhances upto top 25 features and then decreases. Therefore, comparing the performances of all the classifiers to categorize PCOS and non-PCOS patients, the result indicates that, the stacking ensemble model with ‘Gradient Boosting’ classifier as meta learner has outperformed other models utilizing the feature set of top 25 attributes picked using PCA technique. Furthermore, under the observation of expert clinicians, the highly prioritized 25 features selected using the PCA technique were sorted into real-time clinical categories.

## 6 Conclusion

### 6.1 Implications of the study

The methodology presented in this study can be a pioneer in effectively detecting PCOS from patients symptoms and test results through machine learning strategies and thereby can play a potentially beneficial role in improving the reproductive health of thousands of women. The findings of this study can be significantly beneficial towards both patients and healthcare providers in identifying PCOS quickly and efficiently combining the advantages of multiple machine learning classifiers ensembled in one robust model employing minimal number of attributes and thus it is anticipated to be widely used in the real-world clinical practices. The study's outcome can be effectively helpful for the physicians in the arduous task evaluating patients by simplifying the complex diagnostic procedure of PCOS. This computational technique can be deployed in the healthcare facilities of rural areas to detect PCOS autonomously where there is scarcity of expert physicians and resources.

### 6.2 Limitations and future work

Yet, owing to a lack of vast dataset, one of the study's flaws was that it only used machine learning algorithms on a small number of patient data. Real-time data couldn't have been acquired; the dataset was taken from an open source resource. Also, the five traditional ML model categories that have been used as base classifiers in the proposed stacked ensemble model were chosen based on their prominence in this field in earlier studies. The performance might have been different if other types of Ml classifiers had been utilized here. Moreover, the varied number of reduced set of features (35, 30, 25, 20) explored by the feature selection technique have been chosen randomly for this study. In addition, for intelligent clinical applications, explainable AI plays an important role in providing an explanation alongside sufficient justification of AI system predictions, which has not been included in this study. Therefore, the authors hope to investigate more about PCOS detection using larger datasets as well as more types of feature selection techniques in the future incorporating the techniques of eXplainable AI (XAI) with the current study, as well as implement the proposed methodology in other fields of clinical illness predictions.

## Funding statement

This research did not receive any specific grant from funding agencies in the public, commercial, or not-for-profit sectors.

## CRediT authorship contribution statement

Sayma Alam Suha - Conceived and designed the experiments; Performed the experiments.

Muhammad Nazrul Islam, Sayma Alam Suha - Analyzed and interpreted the data; Contributed reagents, materials, analysis tools or data; Wrote the paper.

## Declaration of Competing Interest

The authors declare no conflict of interest.

## Data Availability

Data included in article/supplementary material/referenced in article.
